# OpenKBP-Opt: an international and reproducible evaluation of 76 knowledge-based planning pipelines

**DOI:** 10.1088/1361-6560/ac8044

**Published:** 2022-09-12

**Authors:** Aaron Babier, Rafid Mahmood, Binghao Zhang, Victor G L Alves, Ana Maria Barragán-Montero, Joel Beaudry, Carlos E Cardenas, Yankui Chang, Zijie Chen, Jaehee Chun, Kelly Diaz, Harold David Eraso, Erik Faustmann, Sibaji Gaj, Skylar Gay, Mary Gronberg, Bingqi Guo, Junjun He, Gerd Heilemann, Sanchit Hira, Yuliang Huang, Fuxin Ji, Dashan Jiang, Jean Carlo Jimenez Giraldo, Hoyeon Lee, Jun Lian, Shuolin Liu, Keng-Chi Liu, José Marrugo, Kentaro Miki, Kunio Nakamura, Tucker Netherton, Dan Nguyen, Hamidreza Nourzadeh, Alexander F I Osman, Zhao Peng, José Darío Quinto Muñoz, Christian Ramsl, Dong Joo Rhee, Juan David Rodriguez, Hongming Shan, Jeffrey V Siebers, Mumtaz H Soomro, Kay Sun, Andrés Usuga Hoyos, Carlos Valderrama, Rob Verbeek, Enpei Wang, Siri Willems, Qi Wu, Xuanang Xu, Sen Yang, Lulin Yuan, Simeng Zhu, Lukas Zimmermann, Kevin L Moore, Thomas G Purdie, Andrea L McNiven, Timothy C Y Chan

**Affiliations:** 1 Department of Mechanical and Industrial Engineering, University of Toronto, Toronto, ON, Canada; 2 Vector Institute, Toronto, ON, Canada; 3 Department of Radiation Oncology, University of Virginia Health System, Charlottesville, VA, United States of America; 4 Department of Molecular Imaging Radiation Oncology, UCLouvain, Louvain-la-Neuve, Belgium; 5 Department of Radiation Oncology, Memorial Sloan Kettering Cancer Center, New York, NY, United States of America; 6 Department of Radiation Oncology, The University of Alabama at Birmingham, Birmingham, AL, United States of America; 7 Department of Engineering and Applied Physics, University of Science and Technology of China, Hefei, People’s Republic of China; 8 Shenying Medical Technology Co., Ltd., Shenzhen, Guangdong, People’s Republic of China; 9 Department of Radiation Oncology, Yonsei University College of Medicine, Seoul, Republic of Korea; 10 Department of Physics, National University of Colombia, Medellín, Colombia; 11 Atominstitut, Vienna University of Technology, Vienna, Austria; 12 Department of Biomedical Engineering, Cleveland Clinic, Cleveland, OH, United States of America; 13 Department of Radiation Physics, The University of Texas MD Anderson Cancer Center, Houston, TX, United States of America; 14 Department of Radiation Oncology, Cleveland Clinic, Cleveland, OH, United States of America; 15 Department of Biomedical Engineering, Shanghai Jiao Tong University, Shanghai, People’s Republic of China; 16 Department of Radiation Oncology, Medical University of Vienna, Vienna, Austria; 17 Department of Biomedical Engineering, Johns Hopkins University, Baltimore, MD, United States of America; 18 Department of Radiation Oncology, Peking University Cancer Hospital and Institute, Beijing, People’s Republic of China; 19 Department of Electrical Engineering and Automation, Anhui University, Hefei, People’s Republic of China; 20 Department of Radiation Oncology, Massachusetts General Hospital, Boston, MA, United States of America; 21 Department of Radiation Oncology, University of North Carolina at Chapel Hill, Chapel Hill, NC, United States of America; 22 Department of Medical Imaging, Taiwan AI Labs, Taipei, Taiwan; 23 Department Of Biomedical and Health Sciences, Hiroshima University, Hiroshima, Japan; 24 Medical Artificial Intelligence and Automation (MAIA) Laboratory, Department of Radiation Oncology, The University of Texas Southwestern Medical Center, Dallas, TX, United States of America; 25 Department of Radiation Oncology, Thomas Jefferson University, Philadelphia, PA, United States of America; 26 Department of Medical Physics, Al-Neelain University, Khartoum, Sudan; 27 Institute of Science and Technology for Brain-inspired Intelligence, Fudan University, Shanghai, People’s Republic of China; 28 Studio Vodels, Atlanta, GA, United States of America; 29 Department Computer Science, Aalto University, Espoo, Finland; 30 Department of Electrical Engineering, KULeuven, Leuven, Belgium; 31 Department of Biomedical Engineering, Rensselaer Polytechnic Institute, Troy, NY, United States of America; 32 Tencent AI Lab, Shenzhen, Guangdong, People’s Republic of China; 33 Department of Radiation Oncology, Virginia Commonwealth University Medical Center, Richmond, VA, United States of America; 34 Department of Radiation Oncology, Henry Ford Health System, Detroit, MI, United States of America; 35 Faculty of Health, University of Applied Sciences Wiener Neustadt, Wiener Neustadt, Austria; 36 Competence Center for Preclinical Imaging and Biomedical Engineering, University of Applied Sciences Wiener Neustadt, Wiener Neustadt, Austria; 37 Department of Radiation Oncology, University of California, San Diego, La Jolla, CA, United States of America; 38 Radiation Medicine Program, UHN Princess Margaret Cancer Centre, Toronto, ON, Canada; 39 Department of Radiation Oncology, University of Toronto, Toronto, ON, Canada; 40 Techna Institute for the Advancement of Technology for Health, Toronto, ON, Canada; 41 Department of Medical Biophysics, University of Toronto, Toronto, ON, Canada

## Abstract

**Objective.:**

To establish an open framework for developing plan optimization models for knowledge-based planning (KBP).

**Approach.:**

Our framework includes radiotherapy treatment data (i.e. reference plans) for 100 patients with head-and-neck cancer who were treated with intensity-modulated radiotherapy. That data also includes high-quality dose predictions from 19 KBP models that were developed by different research groups using out-of-sample data during the OpenKBP Grand Challenge. The dose predictions were input to four fluence-based dose mimicking models to form 76 unique KBP pipelines that generated 7600 plans (76 pipelines×100 patients). The predictions and KBP-generated plans were compared to the reference plans via: the dose score, which is the average mean absolute voxel-by-voxel difference in dose; the deviation in dose-volume histogram (DVH) points; and the frequency of clinical planning criteria satisfaction. We also performed a theoretical investigation to justify our dose mimicking models.

**Main results.:**

The range in rank order correlation of the dose score between predictions and their KBP pipelines was 0.50–0.62, which indicates that the quality of the predictions was generally positively correlated with the quality of the plans. Additionally, compared to the input predictions, the KBP-generated plans performed significantly better *P*<0.05; one-sided Wilcoxon test) on 18 of 23 DVH points. Similarly, each optimization model generated plans that satisfied a higher percentage of criteria than the reference plans, which satisfied 3.5% more criteria than the set of all dose predictions. Lastly, our theoretical investigation demonstrated that the dose mimicking models generated plans that are also optimal for an inverse planning model.

**Significance.:**

This was the largest international effort to date for evaluating the combination of KBP prediction and optimization models. We found that the best performing models significantly outperformed the reference dose and dose predictions. In the interest of reproducibility, our data and code is freely available.

## 1. Introduction

**Figure 1.**
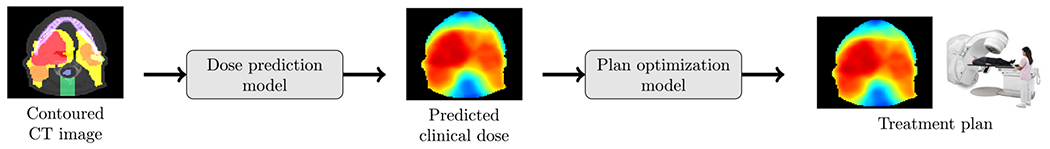
An overview of a complete knowledge-based planning pipeline.

Automated radiotherapy planning is transforming clinical practice and personalized cancer treatment (Moore 2019). The most common type of automated planning is knowledge-based planning (KBP), which leverages knowledge derived from historical clinical treatment plans to generate new treatment plans without human intervention (Cornell et al 2020, Kaderka et al 2021, McIntosh et al 2021). Most common KBP methods are formulated as a two-stage pipeline (see figure 1) that first predicts the dose that should be delivered to a patient (Kearney et al 2018, Nguyen et al 2019) and then converts that prediction into a treatment plan via optimization (Babier et al 2021a, Eriksson and Zhang 2022). Both stages of this pipeline, which are active areas of research, can significantly affect the quality of generated treatment plans (Babier et al 2020). The contributions of this paper are twofold: (1) to provide data that supports KBP optimization research at scale and (2) to establish a connection between dose mimicking (a type of KBP optimization) and conventional planning methods. We expand on the impact of these contributions throughout this paper.

Comparing the quality of competing KBP models from the research community is difficult because the vast majority of research is conducted with large private datasets, as noted in several reviews (Hussein et al 2018, Ge and Wu 2019, Wang et al 2020, Momin et al 2021). To help address this issue, the Open Knowledge-Based Planning (OpenKBP) Grand Challenge was organized to facilitate the largest international effort to date for developing and comparing dose prediction models on a single open dataset (Babier et al 2021b). The OpenKBP dataset, which includes data for 340 patients with head-and-neck cancer who were treated with intensity modulated radiotherapy (IMRT), is limited to dose prediction research (i.e. it is incompatible with KBP optimization research). Although there are still no open datasets for KBP optimization research, there are two open datasets that support research in other areas of plan optimization (Craft et al 2014, Breedveld and Heijmen 2017). However, it is challenging to use these datasets in KBP plan optimization research for two reasons. First, neither dataset includes dose predictions, which are the input to KBP plan optimization models. Second, they are small datasets (123 patients total) that span multiple sites (prostate, liver, and head-and-neck) and multiple modalities (CyberKnife, volumetric modulated arc therapy, proton therapy, and IMRT). While such a diversity in cases is important to demonstrate the robustness and generalizability of optimization algorithms across sites and modalities, this same diversity is a disadvantage when it comes to training dose prediction models, since there is insufficient data for any one site-modality pair (Boutilier et al 2016).

There are several types of KBP optimization models that translate dose predictions into treatment plans. One major type of KBP optimization model is dose mimicking, which generally generates a plan that is similar to an input prediction based on linear (Kierkels et al 2019) or quadratic (McIntosh et al 2017) differences. Another type of KBP optimization model is inverse planning weight estimation, which optimizes patient-specific parameters that make an input dose prediction optimal in a conventional planning model (Chan et al 2014). However, both types of models can also use information beyond a single dose prediction. For example, dose mimicking models can incorporate parameters that reflect the uncertainties in a predicted dose distribution (Zhang et al 2021). Similarly, inverse planning weight estimation models can incorporate an ensemble of dose predictions to leverage the combined wisdom of multiple predictions (Babier et al 2021a). Note that these optimziation models make dose predictions an intermediate step in a KBP pipeline.

Most KBP pipelines are developed as *fully-automated* pipelines that can replace human treatment planners in the planning process (McIntosh et al 2017, Fan et al 2019, Bai et al 2020, Wortel et al 2021). These approaches have demonstrated promising results in prospective research studies where a sizeable portion of KBP-generated plans were considered inferior to human-generated plans, which suggests that there is an opportunity for improvement (Cornell et al 2020, McIntosh et al 2021). In those cases, making manual adjustments to the KBP-generated plan is non-trivial because they are generated by fully-automated pipelines that rely on the quality of the data. In contrast to fully automated pipelines, *semi-automated* pipelines rely on both the quality of data and human expertise, which puts less reliance on the data. For example, a semi-automated KBP pipeline could enable human planners to improve upon a KBP-generated plan via an intuitive process (e.g. inverse planning) and thereby provide a pipeline that leverages both data and human expertise.. In the KBP literature, however, there are relatively few papers that describe tools that humans can intuitively interact with in semi-automated KBP pipeline (Babier et al 2018, Bohara et al 2020, Kaderka et al 2021, Zhang et al 2022).

In this paper, we extend the results from the OpenKBP Grand Challenge with an international validation of 76 KBP pipelines. We made this extension, which we call OpenKBP-Opt, open to provide a benchmark for future KBP optimization research and to lower the barriers for contributing to this research area. We also demonstrate how KBP plan optimization models can be used to initialize a conventional inverse planning process with good patient-specific parameters (i.e. objective weights). This relationship provides a mechanism for transforming some existing KBP optimization models, which are fully-automated pipelines that impede manual intervention, into semi-automated pipelines that promote human planners to improve upon a KBP-generated plan via inverse planning (i.e. a familiar and intuitive process). The data and code to reproduce this paper is publicly available at https://github.com/ababier/open-kbp-opt.

## 2. Materials and methods

**Figure 2.**
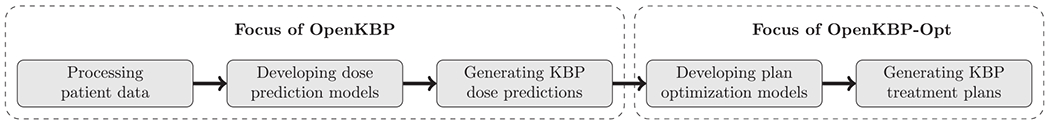
An overview of our methods. Afull description of each component is provided in its corresponding subsection.

Figure 2 separates our methods into five components. The first three components (processing patient data, developing dose prediction models, and generating KBP dose predictions) are based on the results from the OpenKBP Grand Challenge. The final two components (developing plan optimization models and generating KBP treatment plans) are an extension of the OpenKBP Grand Challenge and the focus of this paper. Below, we describe all five components and our analysis.

### 2.1. Processing patient data

We obtained data for 340 patients (*n* = 340) with head-and-neck cancer from the OpenKBP Grand Challenge. The data consisted of a training set (*n* = 200), a validation set (*n* = 40), and a testing set (*n* = 100). The plans were delivered via 6MV step-and-shoot IMRT from nine equidistant coplanar beams at angles 0°, 40°,…, 320°. Those beams were divided into a set of beamlets B, which make up a fluence map. The relationship between the intensity $w_{b}$ of beamlet *b* and dose $d_{v}$ deposited to voxel *v* was determined using the influence matrix $D_{v,b}$ generated by the IMRTP library from the Computational Environment for Radiotherapy Research (Deasy et al 2003) using MATLAB, and it is given by $d_{v}={\sum^{\text{​}}}_{b\in ℬ}D_{v,b}w_{b}$.

### 2.2. Developing dose prediction models

All dose prediction models used in this paper were developed in the OpenKBP Grand Challenge (Babier et al 2021b). During the challenge, teams developed dose prediction models using identical training and validation datasets with access only to ground truth data (i.e. reference dose) for the training set. Every dose prediction model used a neural network architecture that was based on either a U-Net (Ronneberger et al 2015), V-Net (Milletari et al 2016), or Pix2Pix (Isola et al 2017) architecture. Many of the best performing models also used other generalizable techniques like ensembles (Nguyen et al 2021), one-cycle learning (Zimmermann et al 2021), radiotherapy-specific loss functions (Gronberg et al 2021), and deep supervision (Liu et al 2021).

All teams competed to develop models that minimize one of two pre-defined error metrics that quantified the difference between the reference dose and a KBP-generated dose (i.e. their KBP dose predictions). The metrics were: (1) *dose error*, which was the mean absolute voxel-by-voxel difference between two dose distributions, and (2) *dose-volume histogram (DVH) error*, which was the absolute difference between a DVH point from two dose distributions. The DVH error was evaluated on two and three DVH points for each organ-at-risk (OAR) and target, respectively. The OAR DVH points were the ${\text{D}}_{\text{mean}}$ and D_0.1cc_, which was the mean dose delivered to the OAR and the maximum dose delivered to 0.1 cc of the OAR, respectively. The target DVH points were the D_1_, D_95_, and D_99_, which was the dose delivered to 1% (99th percentile), 95% (5th percentile), and 99% (1st percentile) of voxels in the target, respectively. The models were ranked according to: (1) *dose score*, which was the average dose error of a model, and (2) *DVH score*, which was the average DVH error of a model.

### 2.3. Generating KBP dose predictions

In this paper, the OpenKBP organizers collaborated with teams that competed in the OpenKBP Grand Challenge. The 28 teams that completed the final phase of the OpenKBP Grand Challenge were invited to participate in the OpenKBP-Opt project, and 21 of those teams agreed to participate. We obtained dose predictions from the participating teams for each patient in the test set to create a dataset with 2100 dose predictions (21 different predictions for each of the 100 patients). We observed that two models had dose scores that were over two standard deviations (6.3 Gy) above the mean (4.0 Gy), whereas the rest were within half a standard deviation (1.6 Gy) of the mean. Thus, we omitted those two outlier models and proceeded with only 19 KBP models (*n* = 1900 dose predictions).

### 2.4. Developing plan optimization models

Next, we formulated four dose mimicking models, which are a type of KBP optimization model. Each model used the same set of structures and objective functions that are described in sections 2.4.1 and 2.4.2, respectively. However, they differ in how they mimic (i.e. penalize differences) a specific dose distribution. In particular, they each have a different cost function, outlined in section 2.4.3. Note that in this paper the terms *objective function* and *cost function* refer to distinct concepts, and the cost functions in this paper are functions of objective functions.

#### 2.4.1. Structures

All of our optimization models used the same set of regions-of-interest (ROIs) $R_{p}$ for each patient p∈P in our test set. The set $R_{p}$ contained OARs, targets, and optimization structures. The OARs were the brainstem, spinal cord, right parotid, left parotid, larynx, esophagus, and mandible. Each target *t* was a planning target volume (PTV) with a dose level $\theta_{t}$, and those targets were the PTV56, PTV63, and PTV70. The optimization structures were the limPostNeck, which was used to limit dose to the posterior neck, and six PTV ring structures (a 3mm ring and a 6mm ring for each target). These were the same structures used to generate the plans in the original OpenKBP dataset (Babier et al 2021b). Every ROI $r\in R_{p}$ was also divided into a set of voxels $V_{r}$.

#### 2.4.2. Objective functions

Our models used the objective functions in table 1. Each objective function quantified a different measure of the dose delivered to a single ROI $r\in R_{p}$ in a patient p∈P, which we call an objective value. Specifically, the average and maximum dose objective function quantified the average dose and maximum dose delivered to an ROI *r*, respectively. The average dose over and under threshold objective functions quantified the average dose delivered to an ROI *r* that was over and under a dose threshold *f*, respectively. Our average dose over and under threshold objective functions are similar to *tail mean dose* (Romeijn et al 2006) and *conditional value-at-risk* (Rockafellar and Uryasev 2000), which are both defined on the percentiles of a distribution.

In total, we considered 107 objectives functions: seven per OAR, three per target, and seven per optimization structure. The objective functions for each OAR were the mean dose; maximum dose; and average dose over thresholds of *f* equal to 0.25, 0.50, 0.75, 0.90, and 0.975 of the maximum predicted dose to that structure. The objective functions for each target were the maximum dose, the average dose under a threshold *f* equal to the dose level of the target (i.e. $f=\theta_{t}$), and the average dose over a threshold *f* equal to five percent more than the dose level of the target (i.e. $f=1.05\theta_{t}$). The objective functions for each optimization structure were the same as the OAR objective functions. Not all patients had all ROIs, so the models associated with those patients had fewer than 107 objective functions.

#### 2.4.3. Model formulations

Our KBP optimization models performed dose mimicking to generate plans with optimized objective values that closely matched the input objective values from a dose prediction. To streamline our model formulation, let each $m\in M_{p}$ index one of the 107 objective functions (as outlined in section 2.4.2), and let the elements in the vector w represent beamlet intensities $w_{b},\forall b\in B$. Let $g_{m}(w)$ and ${\overset{ˆ}{g}}_{m}$ be objective values of their corresponding objective functions evaluated over the optimized plan and predicted dose, respectively. In all models, the cost functions were formulated such that lower values of $g_{m}(w)$ were favored over higher values. Table 2 presents the cost functions of our dose mimicking models. Each model minimized either the mean or max difference between all corresponding pairs $\left(g_{m}(w),{\overset{ˆ}{g}}_{m}\right)$ of the objective values, which were quantified via an absolute $\left(g_{m}(w)-{\overset{ˆ}{g}}_{m}\right)$ or relative $\left(\left(g_{m}(w)-{\overset{ˆ}{g}}_{m}\right)/{\overset{ˆ}{g}}_{m}\right)$ difference measure, resulting in four dose mimicking models. In the mean difference models, we chose to prioritize the positive differences (i.e. where the optimized plan objective value was higher than the predicted dose objective value) more than the negative differences, which we assigned a small positive weight ϵ (ϵ=0.0001 in our experiments). This was done to incentivize the model to do at least as well as the dose prediction before striving to outperform the dose prediction on other objective functions. In contrast, the max difference models used only a single term because the max difference naturally incentivizes the model to outperform the prediction only once the plan outperforms the prediction across all objective values (i.e. when $g_{m}(w)⩽{\overset{ˆ}{g}}_{m},\forall m\in {ℳ}_{p}$).

The main constraint in all four models was a constraint to limit plan complexity. In particular, the sum-of-positive gradients (SPG) (Craft et al 2007) of all plans generated by the models was constrained to be less than or equal to 65, which was a constraint in the reference plans (Babier et al 2021b). The remaining constraints were simply auxiliary constraints (including auxiliary variables) used to linearize both the objective and cost functions (i.e. the formulations in table 1 and table 2). The optimization models were all formulated in Python 3.7 using OR-Tools 9.1 and solved using Gurobi 9.1 on a single computer with an Intel i7-8700K (6-Core 3.7 GHz) CPU and 16GB of random access memory. Default parameters were used with the Gurobi solver except for *Crossover* set to 0, *Method* set to 2, and *BarConvTol* set to 0.0001, which were selected based on past experience to improve solve time without compromising solution quality.

### 2.5. Generating KBP treatment plans

**Figure 3.**
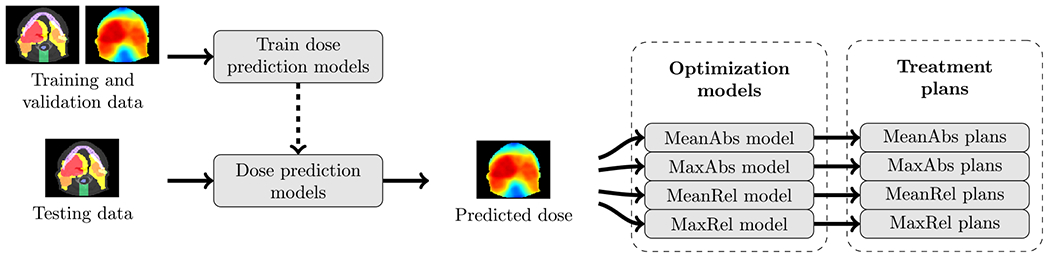
An overview of our process. First, dose prediction models were developed with training and validation data. Second, those models predicted dose for testing data that was used by the dose mimicking models to generate KBP plans.

Next, we assembled 76 KBP pipelines by combining the 19 dose prediction models with each of the four dose mimicking models. Each pipeline was applied to the 100 patients in the testing set, resulting in 7600 KBP plans (see figure 3). We used these plans in our analysis to measure the quality of the respective KBP models. We refer to the plans generated by each dose mimicking model as MeanAbs, MaxAbs, MeanRel, and MaxRel plans.

Altogether, after completing the process in figure 3, we had dose distributions for a set of reference plans (*n* = 100), predictions (*n* = 1900), and KBP plans generated by four dose mimicking models (*n* = 4 × 1900). The reference plans are the plans that were released as part of the OpenKBP Grand Challenge, and the predictions are dose distributions that were submitted by 19 teams in the final testing phase of the challenge. In general, there will be differences between the reference plan, prediction, and KBP plan dose distributions. Differences between a dose prediction and its corresponding KBP plan are due to multiple factors including noisy and undeliverable predictions. Differences between a KBP plan and its corresponding reference plan reflect different trade-offs in the cost function used to generate these plans.

### 2.6. Analysis

We conducted three analyses to measure model performance in terms of dose error, DVH point differences, and clinical criteria satisfaction. We also investigated the theoretical connection between our dose mimicking models and inverse planning. Finally, we summarized empirical optimization metadata.

#### 2.6.1. Dose score and error

We evaluated the KBP models using the dose score and dose error as defined in section 2.2. We calculated the Spearman rank order correlation of the dose score rank between the prediction models and corresponding KBP pipelines. The distribution of dose error was also visualized using a box plot. A one-sided Wilcoxon signed-rank test was used to evaluate whether the dose error of the optimization models was the same (null hypothesis) or lower (alternative hypothesis) than the dose prediction models. For all hypothesis tests in this paper, *P* < 0.05 was considered significant.

#### 2.6.2. DVH point differences

To measure the relative quality of dose distributions from a clinical perspective, we examined the distribution of DVH point differences between the reference and KBP-generated dose. The differences were evaluated over the DVH points listed in section 2.2 and visualized using boxplots. We used the one-sided Wilcoxon signed-rank test to evaluate whether the dose generated by all optimization models performed the same (null hypothesis) or better (alternative hypothesis) than the dose predictions. This test was chosen to evaluate the aggregate performance of all optimization models relative to the predictions. Lower values were better for ${\text{D}}_{\text{mean}}$, D_0.1cc_, and D_1_; higher values were better for D_95_ and D_99_.

#### 2.6.3. Expected clinical criteria satisfaction

As another measure of plan quality, we examined the proportion of clinical criteria that were satisfied by the reference plans and KBP-generated dose. One criterion was evaluated for each ROI (see table 3). The target criteria were evaluated after overlap between targets, which was removed when processing patient data for the OpenKBP dataset, was reinstated. We tabulated the proportion of clinical criteria that were satisfied by the reference plans, dose predictions, MeanAbs plans, MaxAbs plans, MeanRel plans, MaxRel plans, and the plans from the KBP pipeline that satisfied the most clinical criteria overall. We also plotted the proportion of OAR, target, and all ROI clinical criteria that each of the 76 KBP pipelines achieved.

#### 2.6.4. Theoretical analysis of dose mimicking models

To justify our choice of dose mimicking models, we conducted a theoretical analysis into their structure using linear programming duality theory (Bertsimas and Tsitsiklis 1997, Chapter 4). This analysis was based on previous literature that showed a connection between Benson’s method (Benson 1978), which identifies efficient solutions to multi-objective optimization models, and estimating the weights for inverse planning (Chan et al 2014). We were motivated to conduct a similar analysis as in Chan et al (2014) because our dose mimicking models are similar to the formulations in Benson (1978). In particular, we linearized the dose mimicking models, took their duals, and related the dual variables to objective weights ${\overset{ˆ}{\alpha }}_{m}$ in a conventional multi-objective inverse planning problem depicted in model (1):

(1) $$\underset{w}{\text{minimize}}\sum_{m\in {ℳ}_{p}}{\overset{ˆ}{\alpha }}_{m}g_{m}(w),\;\text{subject to}\;\text{SPG}⩽65,$$

Auxiliary constraints to linearize functions in Table 1 and 2.

#### 2.6.5. Optimization metadata

Lastly, we summarized the metadata that each optimization model generated. In particular, we evaluated the average proportion of objective weight that each model assigned to OAR, target, and optimization structure objective functions. We also recorded the average, first quartile, and third quartile solve times.

## 3. Results

In this section, we summarize the performance of the 19 dose predictions models, four dose mimicking models, and 76 KBP pipelines. We also complete our theoretical analysis of dose mimicking models and summarize the metadata generated by our experiments.

### 3.1. Dose score and error

Table 4 summarizes the rank order correlation between the dose prediction models and their corresponding KBP pipelines. We found that the rank of a prediction model was positively correlated with its corresponding KBP pipeline rank. However, there was a wide range in correlation from 0.50 to 0.62. This demonstrates that high quality predictions are correlated with high quality plans, but this result also indicates that a dose prediction model that outperforms a competitor will not always generate better plans when it is used as input to a dose mimicking model. Additionally, the KBP plans generated by an optimization model that evaluated relative differences (i.e. MeanRel and MaxRel) achieved higher rank order correlations than their counterparts that evaluated absolute differences (i.e. MeanAbs and MaxAbs).

**Figure 4.**
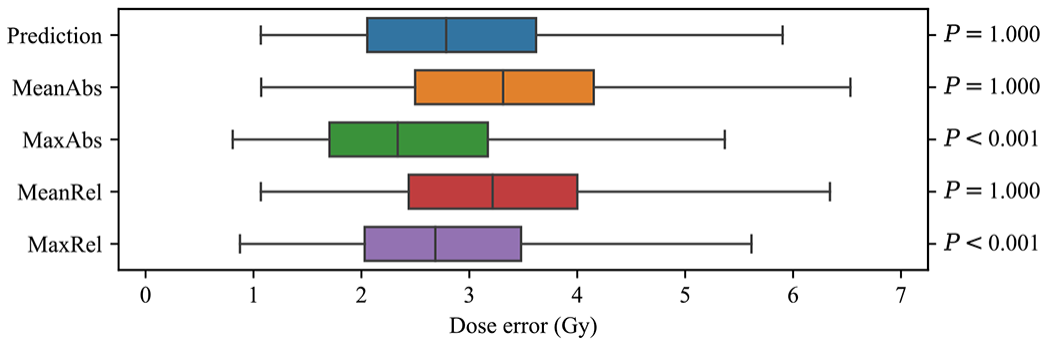
The distribution of dose error over all KBP-generated dose (*n* = 1900 points in each box). Boxes indicate median and interquartile range (IQR). Whiskers extend to the minimum of 1.5 times the IQR and the most extreme outlier.

The dose errors of predictions and KBP plans are shown in figure 4. Two of the four sets of KBP plans (those generated by MaxAbs and MaxRel) had a median dose error that was lower than the median dose error of the predictions (2.79 Gy), implying that it is possible for optimization models to generate dose distributions that more closely resemble the reference plan dose, compared to dose predictions. These two models also achieved a significantly lower error (*P* < 0.001) than predictions. The MaxAbs model achieved the lowest median dose error (2.34 Gy).

### 3.2. DVH point differences

**Figure 5.**
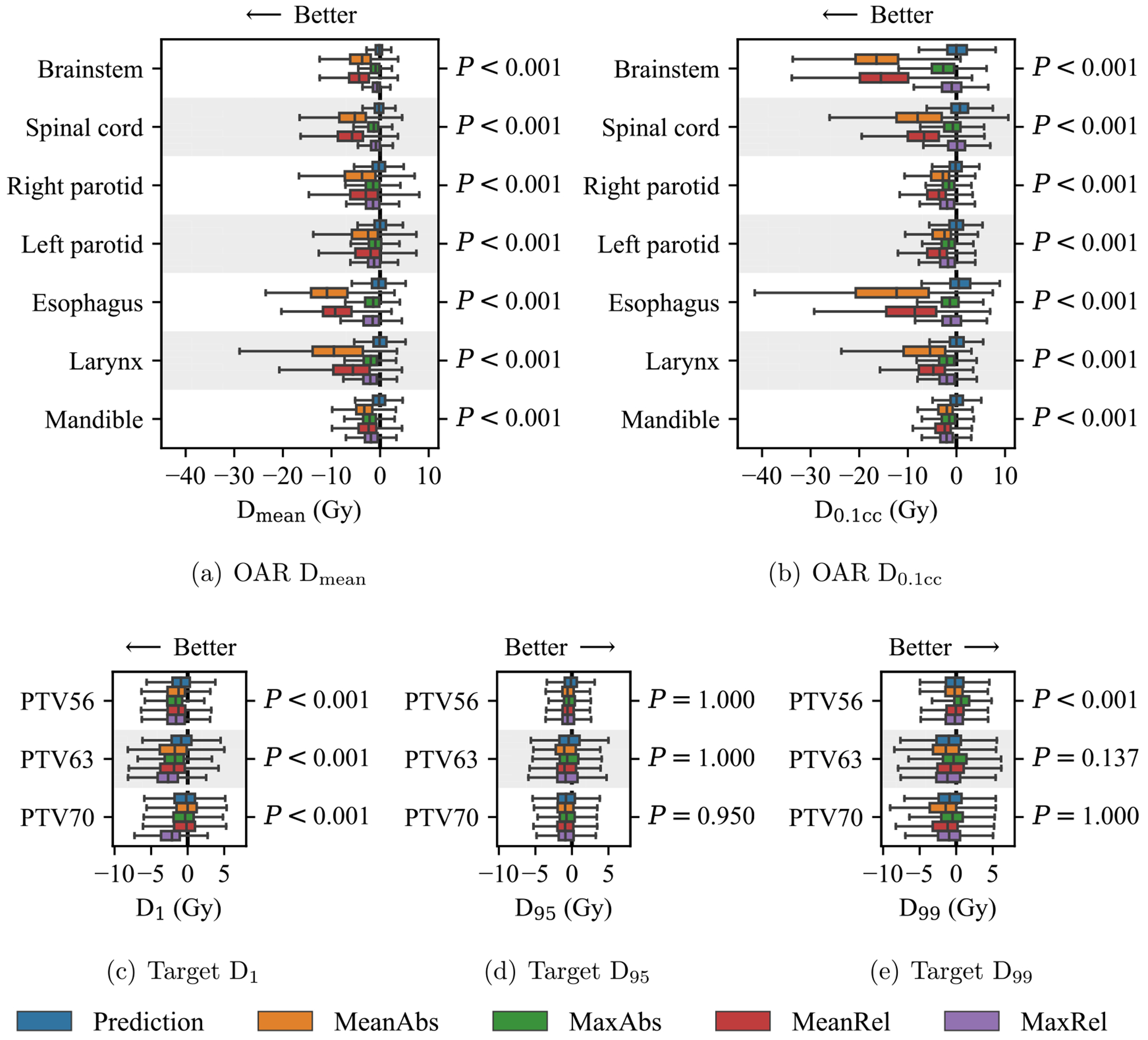
The distribution ofDVHpoint differences between the reference dose and each set of KBP-generated dose. Negative differences indicate cases where the KBP-generated dose had a lower DVHpoint than the reference dose, and arrows indicate the direction where KBP-generated dose is considered better than reference dose for each DVHpoint. Boxes indicate median and IQR. Whiskers extend to the minimum of 1.5 times the IQR and the most extreme outlier.

Figure 5 shows the DVH point differences between the reference dose and KBP-generated dose. In general, dose mimicking tends to produce a plan dose that is significantly better than the dose it received as input from a dose prediction model. In particular, the KBP plan dose is significantly better on 18 of the 23DVH points than the predicted dose (all OAR points and four target points). The five DVH points where the plans were not significantly better are the three D_95_ points and two D_99_ points.

### 3.3. Expected clinical criteria satisfaction

In table 5, we compare the percentage of criteria that were satisfied by the reference plans (*n* = 100), predictions (*n* = 1900), plans generated by each of the four dose mimicking models (*n* = 4 × 1900), and plans generated by the top performing KBP pipeline (*n* = 100). We use the term *baselines* to refer to the reference dose and dose predictions collectively. The top performing KBP pipeline (denoted ‘Best’ in table 5) was defined as the single pipeline (i.e. the combination of one dose prediction model and one dose mimicking model) whose plans satisfied the most clinical criteria. Of all dose mimicking models, the MaxRel and MeanAbs models generated plans that satisfied the fewest (69.8%) and most (72.9%) ROI clinical criteria, respectively. For comparison, predictions only satisfied 66.2% of all clinical criteria, which was 3.5 percentage points lower than the reference plans (69.7%). The best KBP pipeline, which used the MeanAbs model and one of the 19 prediction models (discussed later), satisfied 77.0% of all ROI clinical criteria.

In general, clinical criteria satisfaction varied across each ROI criterion. The brainstem, spinal cord, esophagus, and mandible criteria were each satisfied more than 85% of the time across all the baselines and our dose mimicking models in table 5. The right parotid, left parotid, and larynx were satisfied less than 40% of the time by the the two baselines. In contrast, each of our four dose mimicking models generated a higher average criteria satisfaction for these ROIs compared to the baselines. In fact, some were substantially higher. For example, the average criteria satisfaction of the MeanAbs model on the larynx was 71.5%, compared to an average of 36.2% for the baselines. In aggregate over all 19 prediction models, the performance of the four dose mimicking model was comparable or slightly worse than the reference dose in terms of criteria satisfaction in the targets. However, the best KBP pipeline outperformed the baselines on all criteria.

**Figure 6.**
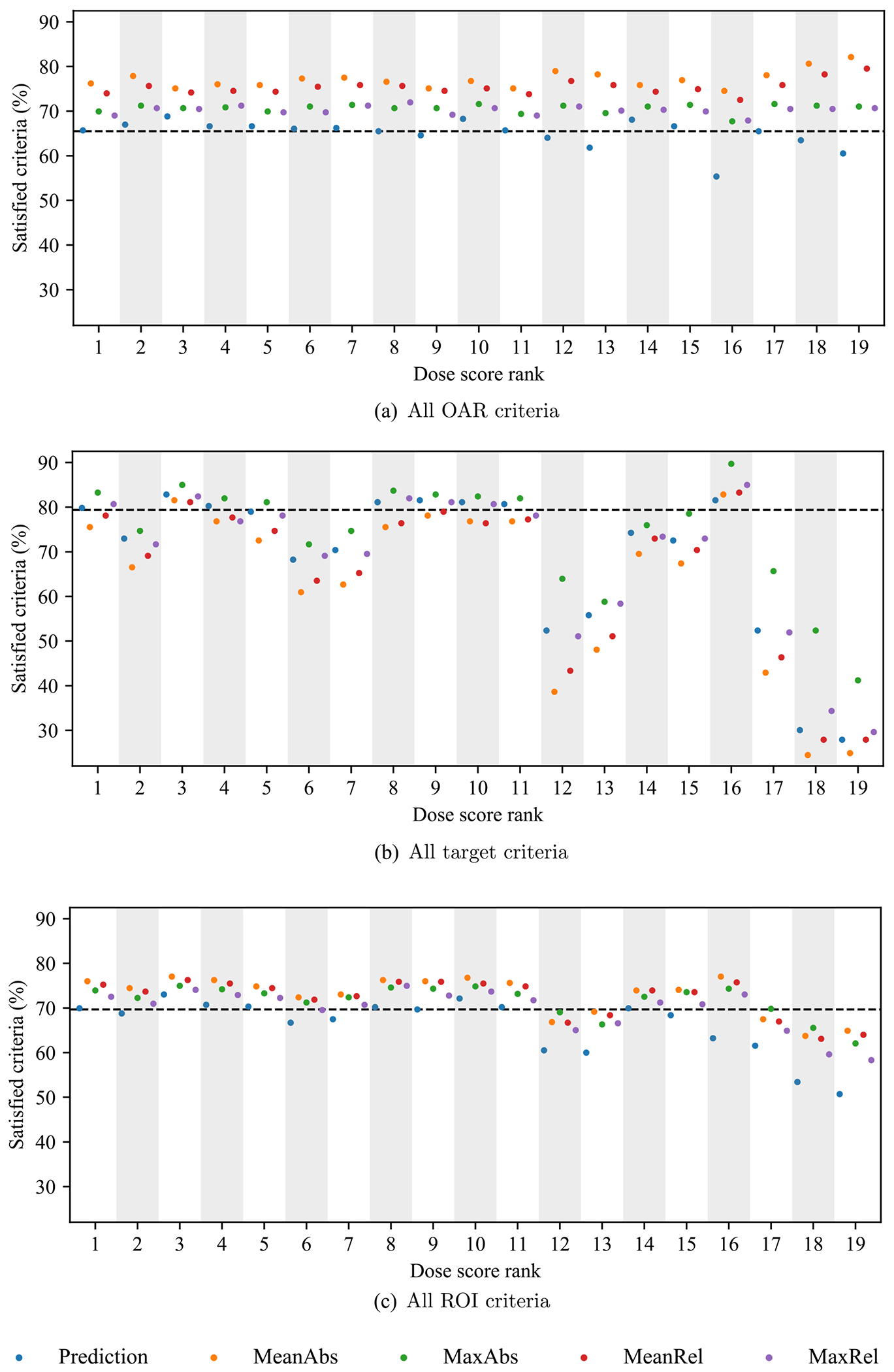
The percentage of all (a) OAR, (b) target, and (c) ROI clinical criteria that were satisfied by each KBP pipeline, which are labeled by their prediction dose score rank. The points indicate the percentage of satisfied criteria for *n* = 100 patients. Adashed line indicates the percentage of criteria satisfied by reference plans.

Figure 6 summarizes the clinical criteria that were satisfied by each of the 76 KBP pipelines that we evaluated. The spread in OAR criteria satisfaction across all 19 models (55.4%–82.1%) was lower than that of target criteria satisfaction (24.5%–89.7%), see figures 6(a) and (b), respectively. Overall, the MeanAbs model generated plans that satisfied more criteria than the other three dose mimicking models for 16 of the 19 dose prediction models (see figure 6(c)). Additionally, the pipelines that used better prediction models (i.e. lower dose score ranks) generally produced plans with higher criteria satisfaction. Interestingly, however, the best performing KBP pipeline (from the last column of table 5) used the dose prediction model that ranked 16th in terms of dose score. Note that the poor performing KBP pipelines used the 12th, 13th, 17th, 18th, and 19th ranked dose prediction models. Since the dose mimicking columns in table 5 included all KBP pipelines, these poor performing models contributed to low performance that was most pronounced on the target criteria. In contrast, many of the KBP pipelines that used the top ranked models prediction models clearly performed much better on target criteria.

### 3.4. Theoretical analysis of dose mimicking models

We use theoretical results from Chan et al (2014) to demonstrate the connection between our dose mimicking formulations and inverse planning. The inverse planning problem presented previously as model (1), is presented again in vector and matrix notation to follow Chan et al (2014). The objective functions are represented as the rows of the matrix C and the objective weights are represented by the vector $\overset{ˆ}{\alpha }$. The decision variables, which include the fluence variables $\left(w_{b},\forall b\in B\right.$) and auxiliary variables, are represented by vector x. The SPG and auxiliary constraints are encoded in the matrix A and vector b. With this vector and matrix notation, we can write the inverse planning problem as model (2):

(2) $$\underset{x}{\mathrm{minimize}}\;{\overset{ˆ}{\alpha }}^{\prime }\mathrm{Cx},\;\text{subject to}\;\mathrm{Ax}=b,\;\;\;\;\;x⩾0.$$

Table 6 presents the formulations of the four dose mimicking models and their respective dual models in vector and matrix notation. The positive and negative differences between optimized objective values Cx and predicted objective values $C\overset{ˆ}{x}$ are represented by vectors σ and δ, respectively. The max difference between the optimized and predicted objective values is expressed as a scalar ζ. The dual variables of the dose mimicking models are denoted by α and p. The vectors of all 0 and 1 are denoted by **0** and e, respectively. The symbol ⊙ denotes element-wise multiplication of two vectors and prime denotes the transpose operator.

Next, we complete our theoretical analysis. We first observe that the weight estimation technique developed in Chan et al (2014) is identical to our dual formulations (see table 6) except for the constraints related to the objective weights α, which prevent trivial solutions to the weight estimation technique. In the context of our models, proposition 5 from Chan et al (2014) establishes that an optimal decision vector $x^{*}$ from each dose mimicking model is also optimal for the inverse planning model with objective weights equal to the optimal dual vector $\alpha^{*}$, which is a byproduct of solving the corresponding dose mimicking model. This result means that the solution to each dose mimicking model is also optimal to an inverse planning model with a particular set of objective weights (i.e. $x^{*}$ is an optimal solution for model (2) when $\overset{ˆ}{\alpha }=\alpha^{*}$). Additionally, by complementary slackness, a plan generated by the MeanAbs or MeanRel model will achieve the same objective values (i.e. ${Cx}^{*}$) as a plan that is optimal for its corresponding inverse planning model. These theoretical results were validated computationally but omitted for brevity.

### 3.5. Optimization metadata

In table 7, we present metadata that was generated by each optimization model, which assigned a different proportion of weight to the objectives for each group of ROIs. The models that evaluate relative differences (i.e. MeanRel and MaxRel) spread the proportion of weight relatively evenly between the OAR and target objectives, but the other two models assigned the majority of the weight to target objectives with no more than 0.018 weight to OARs. Additionally, the optimization structures generally received the smallest proportion of weight with the exception of the MaxAbs model, which assigned more weight to optimization structure objectives (0.170) than OAR objectives (0.011). There was also a wide range in average solve time between the models (222–393 s). On average, the MaxAbs model was the fastest.

## 4. Discussion

KBP research is flourishing. However, optimization models for KBP (e.g. dose mimicking) have received much less attention in the literature than dose prediction models. In this paper, we developed four dose mimicking models and evaluated their performance with 19 different dose prediction models, which were inputs to the optimization models. We showed that both the dose prediction model and optimization model contributed to considerable variation in the quality of plans generated by the corresponding KBP pipeline. Additionally, we conducted a theoretical analysis to show that our KBP optimization models generate plans that are optimal for a multi-objective inverse planning model with particular weights.

Our data and code is published at https://github.com/ababier/open-kbp-opt to enable others to reproduce our results, which meets the gold standard in reproducibility (Heil et al 2021). Our data includes the first open dataset with reference plans and predictions. We hope that this effort produces a common resource and lowers the barriers for future KBP optimization research, given that researchers must currently acquire their own private datasets and develop in-house prediction models before they can start testing new KBP optimization models.

Our open dataset contains the data for 100 patients who were treated with IMRT and a sample of high quality dose predictions for those same patients. The dataset was curated for the purpose of developing new fluence-based KBP optimization models that use ROI masks, dose influence matrices, and dose predictions. The dose predictions were generated by 21 dose prediction models that were developed by an international group of researchers, which provided a diverse sample of realistic inputs for a KBP optimization model. Two of those prediction models (the 20th and 21st ranked models) were removed from our analysis because their dose scores were poor, which we elaborated on in section 2.3. For completeness, however, those 200 predictions are also available as part of our dataset.

We also performed a theoretical analysis to justify our dose mimicking models. Our key theoretical finding was that dose mimicking and conventional inverse planning are equivalent under certain specifications of the objective weights. This allows us to interpret previous weight estimation techniques (Chan et al 2014) through the more intuitive lens of dose mimicking models. Finally, by connecting dose mimicking to inverse planning, there is the potential to convert fully-automated KBP pipelines into semi-automated pipelines. Specifically, we use dose mimicking to generate a high-quality plan with its corresponding objective weights, which reflect the priorities of the input dose prediction, and those objective weights can be used in an inverse planning model (i.e. model (3)). This is advantageous because it enables human planners to improve the quality of plans generated by KBP via a conventional inverse planning process. By enabling this intuitive human interaction, we can create a semi-automated KBP pipeline that is aligned with a common belief that AI will augment, rather than replace, the duties of healthcare practitioners (Ahuja 2019).

Evaluating the performance of optimization models using many different dose predictions helps to identify interaction effects between these two stages of a KBP pipeline (Babier et al 2020). For example, the 16th ranked dose prediction model generated lower quality predictions (in terms of dose error) than most of its competitors. However, when used in a KBP pipeline with the right optimization model, in this case the MeanAbs model, it generated high quality plans that achieved more clinical criteria than any other KBP pipeline. In other words, the errors made by the 16th ranked model that contribute to its low prediction quality were corrected by the KBP optimization model. Note that the 16th ranked prediction model achieved the fewestOAR criteria (55.4%) and the third highest target criteria (81.5%), which suggests that the MeanAbs model was adept at correcting prediction errors related to under and over predictingOAR and target criteria satisfaction, respectively. Since these interaction effects contribute to considerable variation in quality, it is important to evaluate KBP optimization models across a diverse set of dose prediction models. Additionally, if we can understand what types of prediction error are most highly correlated with KBP plan quality we could propose better evaluation metrics to drive KBP prediction research towards making predictions that consistently translate into higher quality plans.

As in the original OpenKBP Grand Challenge, a limitation of this work is that we use synthetic dose distributions (i.e. the reference dose) as a substitute for real clinical dose. Although these dose distributions were subject to less quality assurance than clinical plans, they were previously shown to be of similar quality (Babier et al 2021b). A second limitation of this work is that the dose prediction models were developed with the goal of optimizing the dose andDVH scores. There may be other scoring metrics that are better suited for developing a dose prediction model that excels in a KBP pipeline. This is a possible direction for future research. Lastly, this work only covers a single site and treatment modality. There is no guarantee that KBP optimization models that are developed with this dataset can generalize to other sites or treatment modalities.

## 5. Conclusion

In this paper, we combined the dose predictions contributed by a large international team with several KBP optimization models, resulting in 76 KBP pipelines. This was the largest international effort to date on KBP pipeline evaluation. We found that the best performing pipeline significantly outperformed the baselines. In the interest of reproducibility, our data and code is freely available.

## Figures and Tables

**Table 1.** The formulations for our objective functions.

	Objective function
Average dose	$\underset{v\in V_{r}}{\mathrm{mean}}\left(d_{v}\right)$
Maximum dose	$\underset{v\in V_{r}}{\max}\left(d_{v}\right)$
Average dose over threshold	$\underset{v\in V_{r}}{\mathrm{mean}}{\left(d_{v}-f\right)}^{+}$
Average dose under threshold	$\underset{v\in V_{r}}{\mathrm{mean}}{\left(f-d_{v}\right)}^{+}$

**Table 2.** The cost functions for each dose mimicking model that minimize mean absolute (MeanAbs), max absolute (MaxAbs), mean relative (MeanRel), and max relative (MaxRel) differences between all pairs of the optimized and predicted objective values $\left(g_{m}(w),{\overset{ˆ}{g}}_{m}\right)$.

	Dose mimicking model cost function
MeanAbs	$\underset{m\in M_{p}}{\mathrm{mean}}{\left(g_{m}(w)-{\hat{g}}_{m}\right)}^{+}+\epsilon \underset{m\in M_{p}}{\mathrm{mean}}{\left(g_{m}(w)-{\hat{g}}_{m}\right)}^{-}$
MaxAbs	$\underset{m\in M_{p}}{\max}\left(g_{m}(w)-{\hat{g}}_{m}\right)$
MeanRel	$\underset{m\in M_{p}}{\mathrm{mean}}{\left(\frac{g_{m}(w)-{\hat{g}}_{m}}{{\hat{g}}_{m}}\right)}^{+}+\epsilon \underset{m\in M_{p}}{\mathrm{mean}}{\left(\frac{g_{m}(w)-{\hat{g}}_{m}}{{\hat{g}}_{m}}\right)}^{-}$
MaxRel	$\underset{m\in M_{p}}{\max}\left(\frac{g_{m}(w)-{\hat{g}}_{m}}{{\hat{g}}_{m}}\right)$

**Table 3.** The clinical criteria that we used to evaluate dose distributions.

Structures	Clinical criteria
OARs	
Brainstem	$D_{0.1cc}⩽50.0\;Gy$
Spinal cord	$D_{0.1cc}⩽45.0\;Gy$
Right parotid	$D_{\text{mean}}⩽26.0\;Gy$
Left parotid	$D_{\text{mean}}⩽26.0\;Gy$
Esophagus	$D_{\text{mean}}⩽45.0\;Gy$
Larynx	$D_{\text{mean}}⩽45.0\;Gy$
Mandible	$D_{0.1cc}⩽73.5\;Gy$
Targets	
PTV56	$D_{99}⩾53.2\;Gy$
PTV63	$D_{99}⩾59.9\;Gy$
PTV70	$D_{99}⩾66.5\;Gy$

**Table 4.** Each dose mimicking model is compared to the predictions in terms of Spearman rank order correlation.

	MeanAbs	MaxAbs	MeanRel	MaxRel
Rank order correlation	0.53	0.50	0.62	0.59
Rank order *P*-value	0.019	0.030	0.005	0.008

**Table 5.** The percentage of clinical criteria satisfied in each set of KBP-generated dose. Note that ‘Best’ is defined as the top performing KBP pipeline that generated plans that satisfied the most ROI clinical criteria. The highest percentage of satisfied criteria is bolded in each row.

	Baselines		Dose mimicking models				
	Reference	Prediction	MeanAbs	MaxAbs	MeanRel	MaxRel	Best
OARs							
Brainstem	96.6	97.3	**100.0**	99.5	**100.0**	98.5	**100.0**
Spinal cord	95.5	92.7	99.7	97.3	**100.0**	95.6	**100.0**
Right parotid	32.3	32.7	**46.1**	38.9	45.0	38.0	41.4
Left parotid	30.6	30.1	**43.7**	35.0	41.9	35.0	40.8
Esophagus	93.0	92.7	**100.0**	95.2	**100.0**	97.3	**100.0**
Larynx	37.7	34.7	**71.5**	44.9	58.8	44.6	67.9
Mandible	87.5	89.4	**99.6**	98.7	99.2	99.0	93.1
Targets							
PTV56	91.2	85.8	83.3	91.8	84.1	84.6	**96.7**
PTV63	90.5	86.2	82.2	89.6	84.8	84.8	**92.9**
PTV70	64.0	45.7	37.2	51.6	40.1	47.7	**66.0**
All							
OARs	65.5	65.1	**77.1**	70.6	75.3	70.2	74.5
Targets	79.4	68.7	63.3	74.2	65.3	68.8	**82.8**
ROIs	69.7	66.2	72.9	71.7	72.3	69.8	**77.0**

**Table 6.** The dose mimicking models presented in vector and matrix notation with their dual models. Terms that follow colons indicate the dual variables for that constraint.

	Dose mimicking model	Dual mode
MeanAbs	$\underset{x,\sigma ,\delta }{\mathrm{minimize}}\;{\text{e}}^{\prime }\sigma +\epsilon \;{\text{e}}^{\prime }\delta \;\text{subject to}\;\text{Cx}=\text{C}\hat{x}+\sigma +\delta :\alpha \;\;\;\;\;\text{Ax}=\text{b}\;\;\;\;\;:\text{p}\;\;\;\;\;x⩾0\;\;\;\;\;\sigma ⩾0\;\;\;\;\;\delta ⩽0$	$\underset{\alpha ,p}{\mathrm{minimize}}\;{\text{α}}^{\prime }C\hat{x}-{\text{b}}^{\prime }\text{p}\;\text{subject to}\;C^{\prime }\alpha ⩾A^{\prime }p\;\;:x\;\;\;\;\;\alpha ⩽e\;\;\;\;\;:\sigma \;\;\;\;\;\alpha ⩾\epsilon e\;\;\;\;\;:\delta$

MaxAbs	$\underset{x,\;\zeta }{\mathrm{minimize}}\;\zeta \;\text{subject to}\;\text{Cx}⩽\text{C}\hat{x}+\zeta \text{e}\;:\alpha \;\;\;\;\;\text{Ax}=\text{b}\;\;\;\;\;\;:\text{p}\;\;\;\;\;x⩾0$	$\underset{\alpha ,p}{\mathrm{minimize}}\;{\text{α}}^{\prime }C\hat{x}-{\text{b}}^{\prime }\text{p}\;\text{subject to}\;C^{\prime }\alpha ⩾A^{\prime }p\;:x\;\;\;\;\;\alpha^{\prime }=1\;\;\;\;\;:\zeta \;\;\;\;\;\alpha ⩾0$

MeanRel	$\underset{x,\sigma ,\delta }{\mathrm{minimize}}\;{\text{e}}^{\prime }\sigma +\epsilon \;{\text{e}}^{\prime }\delta \;\text{subject to}\;\text{Cx}=\text{C}\hat{x}⊙(\text{e}+\sigma +\delta )\;:\alpha \;\;\;\;\;\text{Ax}=\text{b}\;\;\;\;\;\;\;\;:\text{p}\;\;\;\;\;x⩾0\;\;\;\;\;\sigma ⩾0\;\;\;\;\;\delta ⩽0$	$\underset{\alpha ,p}{\mathrm{minimize}}\;{\text{α}}^{\prime }C\hat{x}-{\text{b}}^{\prime }\text{p}\;\text{subject to}\;C^{\prime }\alpha ⩾A^{\prime }p\;:x\;\;\;\;\;\alpha ⊙\text{C}\hat{x}⩽\text{e}\;\;:\sigma \;\;\;\;\;\;\alpha ⊙\text{C}\hat{x}⩾\epsilon \text{e}\;\;:\delta$

MaxRel	$\underset{x,\;\zeta }{\mathrm{minimize}}\;\zeta \;\text{subject to}\;\text{Cx}⩽\text{C}\hat{x}⊙(\text{e}+\zeta \text{e})\;:\alpha \;\;\;\;\;\text{Ax}=\text{b}\;\;\;\;\;\;\;:\text{p}\;\;\;\;\;x⩾0$	$\underset{\alpha ,p}{\mathrm{minimize}}\;{\text{α}}^{\prime }C\hat{x}-{\text{b}}^{\prime }\text{p}\;\text{subject to}\;C^{\prime }\alpha ⩾A^{\prime }p\;:x\;\;\;\;\;{\text{α}}^{\prime }C\hat{x}=1\;\;\;:\zeta \;\;\;\;\;\alpha ⩾0$

**Table 7.** A summary of the average proportion of objective weight that was assigned to each group of ROI objectives and the solve time statistics of each dose mimicking model (*n* = 1900 plans in each column).

	MeanAbs	MaxAbs	MeanRel	MaxRel
Objective weight				
OARs	0.018	0.011	0.554	0.417
Targets	0.976	0.819	0.418	0.569
Optimization structures	0.006	0.170	0.028	0.014
Solve time (s)				
Average	389	222	367	393
First quartile	192	107	183	188
Third quartile	502	261	481	507
